# Consumption of Ultraprocessed Foods Among Brazilian Pregnant Women Attended in Primary Healthcare

**DOI:** 10.1155/jnme/4538910

**Published:** 2025-06-02

**Authors:** Gabriele B. Silva, Débora L. F. Silva, Sylvia C. C. Franceschini, Mariana S. Macedo, Claudia C. B. Almeida, Carolina A. Carvalho, Renata J. Pereira, Danielle G. da Silva, Nathalia Pizato, Franciane R. Faria, Naiara Sperandio, Míriam C. R. Barbosa, Anderson M. Navarro, Sandra P. Crispim

**Affiliations:** ^1^Postgraduate Program in Food and Nutrition, Federal University of Paraná, Curitiba, Paraná, Brazil; ^2^Postgraduate Program in Nutrition Science, Federal University of Viçosa, Viçosa, Minas Gerais, Brazil; ^3^Department of Nutrition, Faculty of Biological and Health Sciences, Federal University of Vales Do Jequitinhonha e Mucuri, Diamantina, Minas Gerais, Brazil; ^4^Postgraduate Program in Public Health, Federal University of Maranhão, São Luís, Maranhão, Brazil; ^5^Postgraduate Program in Health Sciences, Federal University of Tocantins, Palmas, Tocantins, Brazil; ^6^Postgraduate Program in Nutrition Science, Federal University of Sergipe, São Cristóvão, Sergipe, Brazil; ^7^Department of Nutrition, University of Brasília, Brasília, Distrito Federal, Brazil; ^8^Faculty of Health Sciences, Federal University of Rondonópolis, Rondonópolis, Mato Grosso, Brazil; ^9^Federal University of Rio de Janeiro, Macaé, Rio de Janeiro, Brazil; ^10^Federal University of Espírito Santo, Vitória, Espírito Santo, Brazil; ^11^Department of Health Sciences, Ribeirão Preto Medical School, University of São Paulo, São Paulo, Brazil

## Abstract

The objective of the study was to describe the dietary intake of Brazilian pregnant women assisted by primary healthcare, focusing on the degree of food processing. Data from the cross-sectional multicenter study of iodine deficiency were analyzed. Participants were selected from basic health units in 11 research centers and completed questionnaires regarding socioeconomic status, demographics, and health. Dietary intake information was collected through 24 h recall and analyzed using GloboDiet software. Descriptive analyses estimated the average energy contribution and confidence interval (%) of NOVA food groups in total energy intake, considering the research center, sociodemographic factors, health status, and pregnancy characteristics. The analysis included 2247 pregnant women without history of thyroid disease or surgery, hypothyroidism, or hypertension. Unprocessed or minimally processed foods accounted for 62.1% of total energy intake, while ultraprocessed foods accounted for 23.8%. Food consumption predominantly occurred at home across all NOVA food groups. Ultraprocessed food consumption was lower during lunch and dinner but higher after dinner and when consumed outside the home, particularly in street settings, markets, parks, and/or beaches, compared with other food groups. This pattern was more prevalent among younger pregnant women (*p* < 0.001), those of white or yellow race (*p*=0.007), residing in urban areas (*p*=0.03), and with higher monthly household income (*p*=0.001). These findings indicate a significant impact on the overall nutritional quality of the diet among pregnant women, with variations based on factors such as age, place of residence, race, income, place of consumption, and occasion of consumption.

## 1. Introduction

The evidence on the association between ultraprocessed food (UPF) consumption and maternal health is growing. According to a meta-analysis of cohort studies, maternal consumption of UPFs was associated to a 48% increased risk of gestational diabetes mellitus and a 28% increased risk of preeclampsia [1]. UPFs have an unfavorable nutritional profile and their consumption negatively affects the macro and micronutrient content of the diet, leading to increased energy density, saturated fat, trans fat, and sugar content, lower fiber and protein content, higher glycemic response, reduced satiety potential, and a greater proinflammatory potential of the diet [2–4]. Thus, reducing the consumption of UPFs can be essential for promoting a healthy diet and is an important target for behavioral change, especially in prenatal interventions [5].

UPFs are also associated with unhealthy eating habits, such as skipping main meals and eating while watching television, driving, or working [6], which characterizes mindless eating [7]. The way we consume food, when, where, and what we do while eating is important in determining satiety, as inattentive eating can lead to a loss of appetite control [6].

If nutritional needs during the gestational period are not met, it can pose risks for women and their newborns, resulting in negative outcomes in infant growth and development and throughout other life cycles [8]. In this sense, the assessment and monitoring of dietary intake are important as they can inform a range of local, regional, national, and international policy decisions aimed at improving the health and wellbeing of this population [9, 10]. By monitoring the dietary intake of pregnant women, policymakers can identify emerging nutritional challenges, such as the rising consumption of UPFs, and implement targeted strategies to address them. These efforts can lead to better health outcomes for both mothers and their children, ensuring that dietary guidelines and public health initiatives are aligned with the actual nutritional needs of this vulnerable population.

According to data from the latest Brazilian National Food Consumption Survey in 2017/2018, a little over half (53.4%) of the calories consumed in Brazil came from unprocessed or minimally processed foods, 15.6% from processed culinary ingredients, 11.3% from processed foods, and 19.7% from UPFs [11]. However, these results did not include a representative sample of pregnant women in the assessment. In fact, there is limited data on the dietary intake of pregnant women with national coverage, especially regarding the consumption of UPFs by this population [12, 13].

Despite limited data on UFP consumption among pregnant women in Brazil, there is growing global interest in understanding how UPF intake affects both maternal and child health. A U.S. study found that 54.4% of pregnant women's energy intake came from UPF, with a 1% increase linked to a 1.33 kg gain in gestational weight [14]. In Spain, UPFs made up 17% of the diet, with sugar-sweetened beverages being the most consumed [15]. Israeli pregnant women consumed 15.6%–43.4% of their energy from UPFs [16], and a Norwegian study linked UPF intake during pregnancy to increased ADHD symptoms in children [17].

Therefore, the aim of this study was to characterize the dietary intake of Brazilian pregnant women receiving prenatal care in primary healthcare, according to the degree of food processing.

## 2. Materials and Methods

### 2.1. Study Design and Location

This was a cross-sectional study conducted using food consumption data from pregnant women participating in the Multicentre Study on Iodine Deficiency (EMDI-Brazil). The study was carried out in 11 municipalities, also referred to as research centers, with at least one center in each macroregion of the country.

The study was conducted in accordance with the guidelines of Resolution no. 466/2012 of the National Health Council and was approved by the Human Research Ethics Committee of the Federal University of Viçosa (coordinating center), under the protocol number no. 2,496,986. All participants provided signed informed consent.

The study population consisted of adult pregnant women (over 18 years old) undergoing prenatal care within the Brazilian Unified Health System (hereafter, SUS) at one of the Basic Health Units (hereafter, UBS). Pregnant women with a history of thyroid disease and/or surgery, self-reported diagnosis of hypothyroidism, pre-existing hypertension, or hypertensive disorders of pregnancy were excluded due to the aims of the multicentre study.

The previously defined sample size for the multicentre study was calculated based on the following parameters: a minimum proportion of 8% iodine deficiency in pregnant women, a relative error of 50% (with a range of 4%–12%), and a confidence level of 95%. These parameters resulted in a simple random sample of 177 subjects per municipality. Since it was a complex sample selected from the family health strategy units, which make up the primary healthcare network in each municipality, the calculation included the effect of the design effect of 1.5, which increased the expected sample size to 266 pregnant women in each research center.

The pregnant women were selected using a stratified sampling plan with two-stage random draw. The primary sampling units were the UBS in the municipalities while the pregnant women receiving care at these UBS served as the secondary sampling units. However, data collection was interrupted in 2020 due to the social distancing measures imposed by the COVID-19 pandemic, which affected Brazilians' access to public health services and limited data collectors' ability to reach pregnant women. Ultimately, the dietary intake of 2247 pregnant women was evaluated.

### 2.2. Data Collection

Data collection began in September 2018 and concluded in March 2020, conducted by interviewers who were trained by the EMDI-Brazil coordination team. Their training included sessions on application of 24 h dietary recalls (R24 h), which included instructional videos for assessing food consumption, available at https://gupea.ufpr.br. Data collection took place in primary health care units while the pregnant women were waiting for or after their appointments and, sometimes, also in the pregnant women's households.

To characterize the sample, social, economic, demographic, health, and lifestyle data were collected from pregnant women using a semistructured questionnaire with the assistance of Research Electronic Data Capture (RedCap) [18].

Pregestational Body Mass Index (BMI) was obtained from the pregnant women's records, and if it was not available, BMI was calculated based on self-reported pregestational weight and height. The BMI values were categorized according to the criteria of the Center for Disease Control and Prevention [19]. The following variables were also considered in the present study: place of residence: urban and rural areas; race/skin color: white, black, yellow, brown (mixed), or indigenous; schooling: no education and elementary school, high school, graduate, or postgraduate; monthly household income (less than R$1000, between R$1000 and R$3000, and greater than R$3000)—In 2018, 1 Brazilian Real (R$) was equivalent to 3.87 US dollars (US$); gestational trimester (first, second, or third); and current smoking and alcohol consumption habits (yes or no).

### 2.3. Data Collection of Food Consumption

Food consumption data were collected using the 24 h dietary recall (R24 h) method, with a paper-based version of the recall specifically developed for EMDI-Brazil, with detailed procedures outlined elsewhere [20]. In brief, the R24 h was adapted to allow for a more detailed classification of foods based on their degree of processing, distinguishing between homemade and industrialized products. In addition, it included a designated section where participants could provide detailed information about recipes, including the ingredients and their quantities, if known. During the administration of the R24 h, conducted face to face by the interviewer, pregnant women were asked to recall all foods and beverages consumed in the previous 24 h and report the meal times, detailing the foods and quantities consumed. The interview was conducted using the multiple pass method (MPM) to standardize data collection and reduce collection errors. The manual of food portion quantification was used to assist in quantifying the portions of consumed foods [21].

It should be noted that the R24 h in this study was adapted to facilitate data entry into the GloboDiet software, which organizes data in the form of facets and systematically applied descriptors to describe foods. In addition to listing the foods previously consumed by pregnant women, the software also captured the meal times, the place and occasion of consumption, whether the food consumed was homemade or industrially processed, whether it had a brand, its type or flavor, the method of preparation, the recipes for each preparation, and finally, the quantities consumed. Specifically, the place of consumption included the following options: home, work (with catering), friends or family's home, restaurants and cafes, school/work (without catering), on the street, markets, parks or beaches, and other unspecified places. As for the occasion of consumption the options were: breakfast, lunch, dinner, morning or afternoon snack, and after dinner.

One R24 h was administered to the entire sample, while a second recall was conducted with a subsample (∼18%) at least one week apart.

### 2.4. Analysis of Food Consumption Data

The food consumption data collected through printed R24 h forms were entered into the GloboDiet software, Data Entry version. GloboDiet is a highly standardized and computer-based 24 h recall program developed, validated, and implemented by the International Agency for Research on Cancer (IARC) [22–24]. The Brazilian version is one of Latin American adaptations of this software [25], tailored specifically to the Brazilian food context. After entering the R24 h data, data quality control was performed by handling inconsistencies flagged by the software or data tabulators. The inconsistencies were treated in a standardized manner, while respecting the specificities of each research center (e.g., unknown quantities were replaced with the center's consumption median for that specific food, except when the same food had been consumed and reported by the respondent in the R24 h form. In that case, the quantity reported in the R24 h form was used for input).

The consumed foods were classified according to the NOVA classification: unprocessed or minimally processed foods, processed culinary ingredients, processed foods, and ultraprocessed foods [26]. In addition, the classification of foods reported by pregnant women in the EMDI-Brazil study followed the specifications outlined in the base document developed by the Food Exposure Research Group of the Nutrition Department at UFPR (Federal University of Paraná), available at https://www.gupea.ufpr.br [27]. Thus, the classification according to the NOVA system was performed with the addition of a fifth category called “uncertainty” to avoid allocating a food item to a specific group when its classification was uncertain due to lack of detailed information in the recall. For example, orange juice not specified by type and/or brand. Next, the initially classified foods under the “uncertainty” category were reclassified into the most likely consumption category, respecting the specificities of each research center. To achieve this, the coordinators of each center were contacted to ensure the appropriate linking of energy information and food classification. For example, in Aracaju, “pão de queijo” (cheese bread) is prepared differently from other centers and, therefore, contact with local researchers from that research center was essential to classify this item as processed. In Curitiba, it was assumed that frozen cheese bread, which may contain food additives, was more commonly consumed, resulting in classifying this item as UPF. Other uncertainties evaluated in collaboration with center coordinators were related to generic reports of certain food types, such as fish, juice, bread, beans, and couscous without further details. For the final classification, foods that were still categorized as uncertain due to a lack of clear consumption patterns in a center (e.g., unspecified cake) were categorized with the lowest possible level of processing, considering the least conservative scenario, as proposed by the European Food Safety Authority (EFSA) for handling uncertainty in dietary exposure assessment [28].

The information regarding the energy value of food items reported by pregnant women in the EMDI-Brazil study was obtained from the Brazilian Table of Food Composition [29]. Missing data or traces in the composition of foods were treated as zero. Both the linking of data on food consumption with calorie information and the classification of foods according to the NOVA system were conducted at a disaggregated level of individual foods and recipe ingredients, involving two researchers.

The eligibility of the 24 h dietary recall (R24 h) was assessed based on extreme values in daily food group and nutrient consumption, as well as the number of reported food items. R24 h with energy intake below 500 or above 4000 kcal/day, and those with less than five reported foods, were scrutinized for biological plausibility. If a plausible explanation was provided, such as low intake due to pregnancy-related nausea and/or vomiting, or excessive appetite on atypical days, they were deemed eligible and included in the dataset. Conversely, R24 h without explanations were excluded (*n* = 52). The study analyzed food consumption data from 2247 pregnant women, with 18.3% of cases having a R24 h replicated.

The usual energy intake of pregnant women was adjusted for intraindividual variability using the NCI method macros in SAS on demand for academic's version. Distributions were modeled for the group of pregnant women as a whole using the Amount model [30]. Descriptive analyses were performed to characterize food consumption according to the degree of food processing. To describe the data, further classification was performed using the Food and Agriculture Organization/World Health Organization (FAO/WHO) classification available at the FAO/WHO GIFT platform [31]. The contribution (%) of NOVA food groups to total energy intake was evaluated using the proportion means method [32]. The contributions of energy intake according to the NOVA classification by occasion of consumption and place of consumption were estimated and presented in graphs. Point estimates of this contribution and their respective 95% confidence intervals (95% CIs) were calculated for the total sample and by research center, sociodemographic characteristics of the pregnant women and by temporal aspects of data collection: season (winter, summer, autumn, and spring), and day of the week (Monday–Friday vs. Saturday and Sunday). We used independent *t*-tests to compare the mean percentages of food group consumption (classified by NOVA) across two-level categorical variables, such as smoking status. When the data were not normally distributed, the Mann–Whitney *U* test was applied instead. For categorical variables with more than two levels, we used one-way ANOVA to identify significant differences in means or the Kruskal–Wallis test when normality assumptions were violated. If ANOVA showed significant results, Tukey's post hoc test was performed to identify specific group differences. All analyses were performed using SPSS software Version 22.

## 3. Results

**Figure 1:**
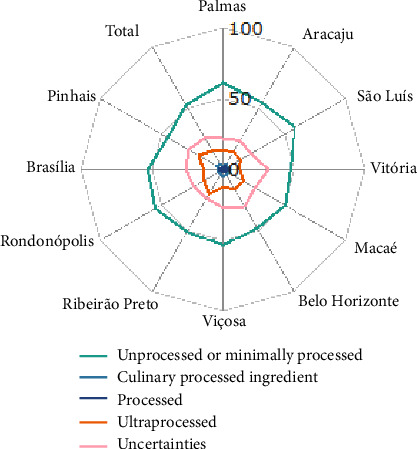
Average contribution (%) of each NOVA classification food groups (including the group of uncertain foods) to total energy intake of pregnant women, according to research center, EMDI-Brazil, 2021.

Approximately half of the calories (52.6%) consumed by pregnant women came from the intake of unprocessed or minimally processed foods, 4.3% from processed culinary ingredients, 2.1% from processed foods, 15.2% from UPFs, and 25.9% from foods initially classified in the “Uncertainty” category. There was some variation in uncertainty estimates among the research centers (Figure 1), with a lower proportion in Palmas (22.0%) and a higher proportion in Vitória (32.0%).

The most commonly classified uncertain foods included unspecified items such as bread, pasta, juices, biscuits, meatballs, jams, tomato sauce, cheese, and cakes. For each of these, an ultraprocessed industrial equivalent was identified in the Brazilian market, along with their minimally processed or processed alternatives.

After the reclassification of foods from the “Uncertainty” category, 62.1% of the ingested calories came from the consumption of unprocessed and minimally processed foods, 4.3% from processed culinary ingredients, 9.8% from processed foods, and 23.8% from UPFs. For center-specific results, refer to Supporting Table 1S, which presents the average contribution (%) of each NOVA food group to the total energy intake of pregnant women by research center.

Regarding the foods that comprised the NOVA classification groups, we identified that meats (16.6%), rice (10.6%), and fruits (4.8%) contributed to approximately one third (32%) of the calories provided by the group of unprocessed and minimally processed foods. In terms of processed culinary ingredients, vegetable oils and fats (2.7%) and sugars (1%) were the foods that predominantly contributed to the energy intake of this group. As for processed foods, the items that had the highest contribution were bread and other wheat products (7.5%), cheeses (1.4%), and processed meats (0.5%), while for the group of UPFs, the foods that contributed the most were sweets and sugars (7%), processed meats (3.0%), and savory snacks (2.8%) (Table 1).

When analyzing the energy contribution of the NOVA classification among pregnant women, certain trends were observed based on demographic characteristics. Pregnant women aged over 35 had lower consumption of UPFs (21.8%; 95% CI: 19.4–24.1) compared with those under 20 (27.4%; 95% CI: 24.7–30.1; *p* < 0.001). Urban residents showed lower intake of unprocessed/minimally processed foods (61.9%; 95% CI: 61.1–62.8; *p*=0.02) and higher intake of UPFs (23.9%; 95% CI: 23.0–24.7; *p*=0.03) compared with rural residents. Pregnant women of Black, mixed race, or indigenous ethnicity consumed more unprocessed/minimally processed foods (63.5%; 95% CI: 62.4–64.6; *p*=0.001) and less processed/ultraprocessed foods (22.5%; 95% CI: 21.5–23.5; *p*=0.007) compared with White/Asian women. Those with lower household incomes had higher consumption of unprocessed/minimally processed foods (below R$1000, 63.2%; 95% CI: 61.6–64.8; *p*=0.003) while higher-income groups had higher intake of UPFs (above R$3000, 26.5%; 95% CI: 24.7–28.4; *p*=0.001). Differences were not significant for schooling, household head, trimester, smoking, alcohol use, or season. Consumption of UPFs was slightly higher on weekends (25.4%; 95% CI: 22.4–28.5) than weekdays (23.8%; 95% CI: 23.1–24.6) although the difference was not statistically significant (Table 2).

**Figure 2:**
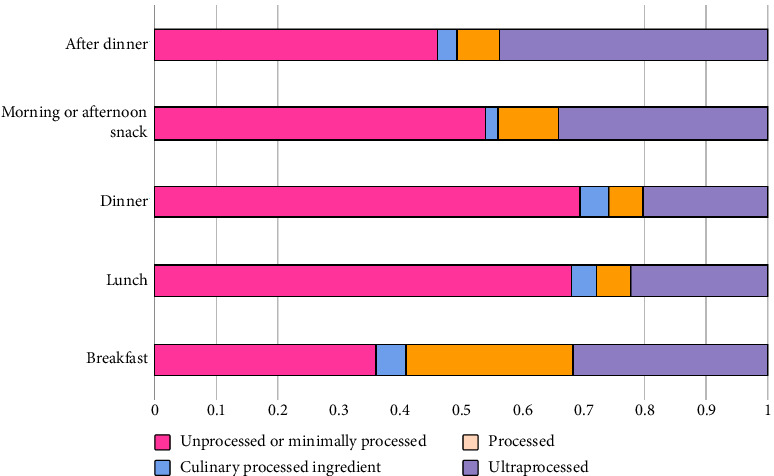
Average contribution (%) of each NOVA classification food groups to total energy intake of pregnant women, according to consumption occasion, EMDI-Brazil, 2021 (*n* = 2247).

**Figure 3:**
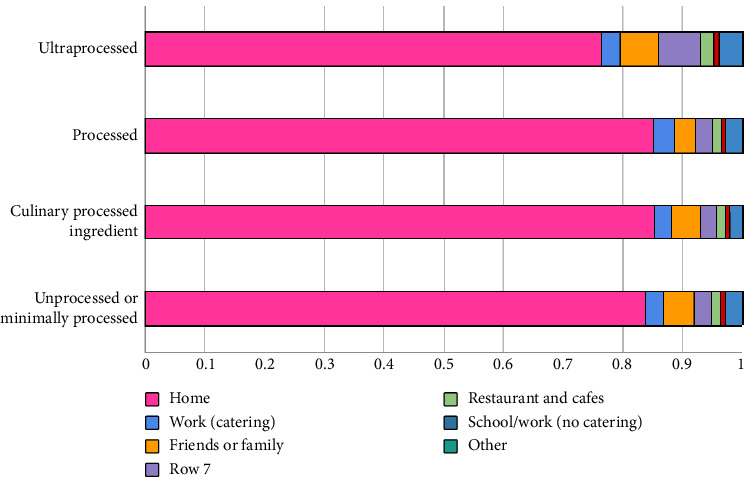
Average contribution (%) of each NOVA classification food groups to total energy intake of pregnant women, according to the place of consumption, EMDI-Brazil, 2021 (*n* = 2247).

In terms of consumption occasions (Figure 2), “breakfast” had the highest energy contribution from processed foods. Conversely, “lunch” and “dinner” had the lowest consumption of UPFs, with over 70% of the energy coming from unprocessed/minimally processed foods and processed culinary ingredients. Ultraprocessed foods accounted for about 30% of the energy intake during breakfast, morning snack, and afternoon snack, with the after-dinner occasion showing the highest percentage around 40%. Finally, home consumption prevailed across all NOVA food groups, with percentages exceeding 70% (Figure 3). Nevertheless, out-of-home consumption of UPFs was notably higher compared with other groups.

## 4. Discussion

The present study described the food consumption of Brazilian pregnant women undergoing prenatal care in primary health care, according to the degree of food processing and the food context. The results showed that the group of unprocessed and minimally processed foods, along with processed culinary ingredients and processed foods, still are the basis of their diet. However, UPFs contributed significantly to the total energy intake of pregnant women (23.8%). Moreover, the consumption of UPFs was lower during lunch and dinner and higher after dinner, especially among younger pregnant women of White or yellow ethnicity, residing in urban areas, and with higher monthly household income. Consumption at home was predominant for all food groups according to the NOVA classification. Nevertheless, the consumption of UPFs was higher out of home compared with other food groups.

The contribution of UPF consumption to energy intake was slightly higher than the national average reported in the POF 2017/2018 (19.7%). However, it is similar to the contribution (20.9%) found by Mariano et al. [33] with data from a nonrepresentative sample of pregnant women participating in this national survey and by Graciliano, Silveira, and Oliveira in a study carried out in Maceio, Brazil (22.2%) [34]. In contrast, our contribution falls below to other Brazilian studies conducted on pregnant women in Rio de Janeiro (35.3%) and Ribeirão Preto (32.1%). Globally, the contribution of UPFs to energy intake observed in this study is lower than that reported for pregnant women in the United States [14] (54.4%) and Mexico [35] (28.0%) but higher than the levels found in Spain [15] (17%) and Norway [36] (14.1%).

Several political, environmental, social, economic, cultural, and biological factors can influence the food consumption of by pregnant women, which may explain the differences in the contribution of UPFs to total energy intake observed between our study and other national and international studies [37]. Despite these variations, Ruiz et al. [13] found that the contribution of UPF consumption was similar between pregnant women (94.8%) and women of reproductive age (90.4%) in Brazil, which reinforces the need for interventions aimed at promoting and ensuring healthy eating for pregnant women and women of reproductive age in the country.

In relation to the centers, it was found that the consumption of UPFs was lower in Aracaju, representing 19.9% of the calories consumed by pregnant women, and higher in Pinhais, representing 29.1% of the calories consumed; a result higher than that of the 2017-2018 POF [11]. This finding is consistent with the literature, which found that the prevalence of UPF consumption was higher, especially in the southern region of the country [33, 38].

In Mariano's study [33], the items that contributed the most to the total energy consumption of the group of unprocessed or minimally processed foods were rice, beef, beans, poultry, fruits, and vegetables, while ready-to-eat or semiready meals, margarine, salted biscuits, and chip-type salty snacks were the items with the highest contribution to UPFs. Our findings are similar for unprocessed or minimally processed foods but not for UPFs, where sweets and sugars, processed meats, and snacks were the foods with the highest contribution.

It should be emphasized, however, that although the consumption of unprocessed or minimally processed foods is predominant, the consumption of rice and beans, two traditionally Brazilian foods, has decreased between the last two national surveys (POF 2008-2009 and POF 2017-2018). The consumption of beans decreased from 72.8%–60% between 2008 and 2018, while the frequency of rice consumption varied from 84% to 76.1% in the same period. Therefore, although these two foods are still the basis of the Brazilian diet, it is noteworthy that they have been losing space on the plates of the Brazilian population [11] and, therefore, it is necessary to monitor their consumption, as they are traditional staple foods in Brazil and their consumption is associated with a healthy diet [39].

Regarding the characteristics of pregnant women, it was observed that the contribution of the UPF group was lower among older pregnant women, as also observed in other studies [40, 41]. This can be explained by the fact that younger individuals, including pregnant women, appear to be exposed to an unhealthy dietary pattern characterized by high levels of total fat, saturated fats, sugar, salt, and a lack of complex carbohydrates, fiber, and micronutrients. This dietary pattern differs from that of older pregnant women, highlighting the vulnerability of the younger group to increased exposure and ease of access to these types of food [42, 43].

Pregnant women living in urban environments seem to have worse food quality in late pregnancy, as pregnant women residing in urban areas have lower consumption of unprocessed and minimally processed foods and higher consumption of processed culinary ingredients and UPFs. In fact, urbanization brings a lot of changes in dietary habits due to lack of time for food preparation and shopping, leading to a preference for more convenient and ready-to-eat foods, as well as meals eaten out of home and replacing regular meals with snacks [44]. This situation appears to be explained by both the higher income levels of residents in these areas and the greater availability of restaurants and food services that facilitate eating out [45].

The highest consumption of unprocessed or minimally processed foods and the lowest consumption of UPFs among pregnant women of black, brown, or indigenous ethnicity was also observed in the study by Costa et al. [46]. According to the authors, these findings may be associated with the socioeconomic and demographic conditions of the population under study, given that dietary consumption is influenced by various factors, including cultural characteristics, food availability, and access [38]. Furthermore, pregnant women receiving primary healthcare and with lower monthly household income showed higher consumption of unprocessed or minimally processed foods, while pregnant women from the same group with higher monthly household income showed greater consumption of UPFs, as seen in other studies [33, 42]. The relationship between income and increased consumption of UPFs has been demonstrated in other studies as well. It is acknowledged that a dietary pattern based on UPFs is still more expensive than a pattern based on unprocessed or minimally processed foods in Brazil [45, 47].

In relation to the days of the week, it was observed that the consumption of UPFs during weekends was slightly higher than on weekdays, as in other studies, since during the weekend, there is usually a significantly higher intake of energy and sugar compared to weekdays, indicating that weekend diet is commonly calorie rich and of low quality [48, 49].

Furthermore, the higher consumption of UPFs after dinner can be explained by the habit of snacking between meals, which is associated with a pattern of consuming calorie-dense foods such as desserts, candies, chocolates, sweetened beverages, snacks, and cookies [50]. In fact, main meals such as breakfast, lunch, and dinner tend to have better nutritional quality when compared with snacks [51].

It is also worth noting that there is a higher consumption of UPFs, such as soft drinks and fast food, out of home compared with other groups in the NOVA classification. This can be partly attributed to the characteristics of these foods, such as their widespread availability, convenience, marketing appeal, and ease of transportation and storage, allowing for consumption anywhere. Therefore, actions and strategies aimed at promoting and encouraging environments that offer healthier food choices outside the home should be consistently prioritized [41].

The consumption of UPFs has negatively impacted the quality of the diet of Brazilian pregnant women, which could lead to increased oxidative stress and affect pregnancy outcomes, as well as being associated with an increased risk of overweight or obesity in the offspring [35, 52]. As UPFs play a larger role in the diet, there is a decrease in the nutritional quality of the diet [34]. Therefore, given the evident growing influence of the consumption of these foods in Brazil, there is a need for interventions regarding the promotion of healthy eating, especially among pregnant women. Based on our study's findings, targeted interventions should focus on key demographics. For instance, specific dietary programs could be developed for younger pregnant women, as they may be more susceptible to the influence of UPF as well as urban residents, who often face greater exposure to fast food and convenience products. In this sense, Brazilian healthcare professionals must be well informed about current nutritional recommendations, including the NOVA classification of foods, to effectively communicate these guidelines to women of reproductive age, during pregnancy, and postpartum. Furthermore, to further understand the long-term impact of these dietary changes on the population, future studies should focus on conducting longitudinal research to observe and evaluate the effects on maternal and child health over time.

As potentialities of the study include the use of an adapted R24 h, following the multiple pass method, with the assistance of the manual of food portion quantification as well as the quality control of food consumption data carried out to minimize errors in food consumption estimates. It is also worth highlighting the collection of a second R24 h in 18.3% of pregnant women, allowing for adjustment of intraindividual variability. Furthermore, the novelty of the work should be emphasized, as it is the first Brazilian study with a national approach to characterize the diet of pregnant women assisted in primary healthcare.

Nevertheless, the study has some limitations. First, self-reported food consumption introduces potential bias, as memory limitations might hinder accurate descriptions of foods consumed [53], potentially compromising results. However, the selection of the R24 h recall as an open and concise method for investigating dietary intake enables both quantitative and qualitative evaluation of several dietary aspects, such as UPF consumption, at both the individual and population levels [54]. Second, lower levels of education could lead to challenges in defining and quantifying food intake. Third, the study was conducted during the COVID-19 pandemic, which likely influenced dietary habits. Lockdowns and social distancing disrupted access to food, increased reliance on processed and convenience foods, and caused changes in eating behaviors due to stress or financial challenges. The pandemic also affected data collection, and restrictions may have limited face-to-face interactions and reduced the sample size in some centers. Lastly, the EMDI-Brazil study was primarily designed to assess the iodine nutritional status of Brazilian pregnant women receiving prenatal care through the SUS. Although the sample selection was based on iodine deficiency, we believe this does not introduce a significant bias that would impact the general dietary patterns observed, including UPF consumption. Moreover, since 71.5% of Brazilian pregnant women received prenatal care through SUS in 2019 [53], it is expected that the sample can be considered largely representative of the broader population.

To end, the NOVA classification categorizes foods based on the extent and purpose of industrial processing and has been widely used as a tool for comparing data across studies and countries. However, its accuracy depends heavily on the level of detail provided by interviewers, as well as the information gathered by researchers (e.g., brand names and food sources) or the chosen dietary assessment method, which can make the evaluation process challenging [54]. In our study, we aimed to maintain transparency in the classification of foods reported by pregnant women and adapt classifications to reflect the regional context of the research centers. Although complete elimination of uncertainty was not possible, we believe these efforts helped reduce biases in classifying foods with uncertain categorization. For future research, we recommend conducting sensitivity analyses when using the NOVA classification to assess UPF consumption, as this can help evaluate the impact of uncertainties on the results as also demonstrated Martinez-Steele et al.'s procedure [55].

## 5. Conclusions

Approximately 52.6% of the calories consumed by the studied pregnant women came from unprocessed or minimally processed foods, while 4.3% came from processed culinary ingredients, 2.1% from processed foods, 15.2% from UPFs, and 25.9% from foods in the “Uncertainty” category. The proportion of uncertainty varied across research centers, with Palmas at 22.0% and Vitória at 32.0%. UPF consumption was influenced by factors such as age, place of residence, race, income, place of consumption, and occasion of consumption. By considering these factors, efforts can be made to reduce UPF consumption and improve the health and nutrition outcomes of pregnant women. This research contributes to monitoring dietary intake among this population, providing valuable data for healthcare improvement, as this information was not previously available.

## Figures and Tables

**Table 1:** Average energy intake of pregnant women, according to the NOVA food group classification of foods, EMDI-Brazil, 2021 (*n* = 2247).

Food groups⁣^∗^	Average kcal/day	% Energy
Unprocessed or minimally processed foods⁣^∗∗^	1123.8	62.1
Meats	300.0	16.6
Rice	192.2	10.6
Fruits	86.1	4.8
Milk and dairy products (excluding cheese)	81.8	4.5
Cakes and sweets	75.2	4.2
Pulses	66.9	3.7
Vegetables	54.5	3.0
Pasta and flours	46.5	2.6
Natural fruit juice	32.2	1.8
Corn products (flakes, cornmeal, etc.)	31.8	1.8
Eggs	30.9	1.7
Tubers	29.6	1.6
Potatoes	22.9	1.3
Fish and seafood	20.6	1.1
Water, tea, coffee	17.7	1.0
Others	35.0	1.9
Processed culinary ingredients	77.8	4.3
Vegetable oils and fats	48.0	2.7
Sugars	17.8	1.0
Animal oils and fats	11.4	0.6
Corn starch	0.6	0.03
Vinegar	0.1	0.01
Processed foods	177.3	9.8
Bread	134.9	7.5
Cheeses	24.7	1.4
Processed meats	9.9	0.5
Others	7.9	0.4
Ultra-processed foods	430.7	23.8
Sweets	127.5	7.0
Processed meats (e.g., ham and sausages)	53.59	3.0
Savory snacks (e.g., croissant and deep-fried Brazilian snack)	49.97	2.8
Ultraprocessed bread and pasta	41.77	2.3
Carbonated soft drinks	34.26	1.9
Composite dishes (e.g., ultraprocessed finger foods)	25.55	1.4
Dairy products	24.3	1.3
Margarines	10.71	0.6
Crisps and curls	13.32	0.7
Condiments (e.g., ketchup and stock cube)	10.22	0.6
Fruit drinks industrialized	10.11	0.6
Industrialized fries	8.81	0.5
Cheese products	5.34	0.3
Corn products	4.39	0.2
Savory pies	2.85	0.2
Others	8.01	0.4

⁣^∗^Based on the Food and Agriculture Organization/World Health Organization classification [31].

⁣^∗∗^NOVA classification [26] was conducted at the food item level. Unprocessed food groups do not include their processed or ultraprocessed equivalents. For example, cakes and sweets classified in this group are intended to reflect their original preparation from minimally processed ingredients (e.g., banana cake) rather than commercially processed versions.

**Table 2:** Average contribution (%) of each NOVA classification food groups to total energy intake of pregnant women, according to sociodemographic, health, gestational characteristics, and temporal aspects of data collection^1^, EMDI-Brazil, 2021.

Characteristics	*N* (%)	Unprocessed or minimally processed foods (%)		Processed culinary ingredients (%)		Processed foods (%)		Ultraprocessed foods (%)	
		Mean	CI	Mean	CI	Mean	CI	Mean	CI
Maternal age (in years)									
Less than 20	219 (9.8%)	59.2	56.4–61.9	3.4^a^	2.9–3.8	10.1	8.7–11.5	27.4^a^	24.7–30.1
20–35	1742 (78.0%)	62.5	61.5–63.4	4.3^b^	4.1–4.5	9.7	9.2–10.3	23.5^a^	22.6–24.4
More than 35	271 (12.1%)	62.8	60.4–65.3	5.1^b^	4.4–5.7	10.3	8.8–11.8	21.8^b^	19.4–24.1
Place of residence									
Urban	2098 (94.5%)	61.9^a^	61.1–62.8	4.4^a^	4.1–4.6	9.8	9.4–10.3	23.9^a^	23.0–24.7
Rural	122 (5.5%)	66.5^b^	63.1–69.8	3.7^b^	3.1–4.2	9.9	7.8–12.1	20.0^b^	16.8–23.1
Race/skin color self-reported									
White or yellow	939 (42.3%)	60.4^a^	59.1–61.7	4.4	4.1–4.7	10.0	9.3–10.7	25.2^a^	23.9–26.5
Brown (mixed), Black, and indigenous	1283 (57.7%)	63.5^b^	62.4–64.6	4.3	4.0–4.5	9.7	9.1–10.3	22.5^b^	21.5–23.5
Schooling									
No education and elementary school	494 (22.3%)	62.6	60.6–64.5	4.4	4.0–4.8	10.7	9.5–11.8	22.4	20.7–24.1
High school	1361 (61.6%)	62.0	60.9–63.0	4.2	4.0–4.5	9.8	9.2–10.4	24.0	23.0–25.0
Graduate or postgraduate	356 (16.1%)	62.5	60.5–64.6	4.5	4.0–5.1	9.1	8.0–10.3	23.8	21.9–25.7
Head of the household									
Herself	560 (26.7%)	62.2	60.6–63.8	4.3	3.9–4.6	10.0	9.2–10.9	23.5	22.0–25.0
Another relative	518 (24.7%)	61.1	59.4–62.8	4.2	3.8–4.6	9.9	8.9–10.9	24.8	23.1–26.5
Partner	1020 (48.6%)	62.7	61.5–63.9	4.4	4.1–4.7	9.7	9.0–10.4	23.2	22.1–24.4
Monthly household income (in income brackets)									
Less than R$^2^1000	605 (30.7%)	63.2^a^	61.6–64.8	4.3	4.0–4.6	10.6	9.7–11.5	21.9^a^	20.5–23.3
Between R$1000 and R$3000	987 (50.1%)	62.6^a^	61.4–63.8	4.2	3.9–4.5	9.5	8.9–10.2	23.7^ab^	22.5–24.8
Greater than R$3000	377 (19.1%)	59.4^b^	57.5–61.3	4.6	4.0–5.2	9.5	8.4–10.5	26.5^b^	24.7–28.4
Gestational trimester									
First	505 (22.7%)	60.5	58.7–62.3	4.3	3.9–4.7	10.0	9.0–11.0	25.2	23.5–26.9
Second	836 (37.5%)	62.2	60.9–63.6	4.5	4.2–4.9	9.9	9.1–10.7	23.3	22.1–24.6
Third	887 (39.5%)	63.1	61.8–64.5	4.1	3.8–4.4	9.7	9.0–10.4	23.0	21.7–24.3
Current smoking									
Yes	103 (4.6%)	63.4	58.8–68.0	3.8	3.1–4.6	8.6	6.5–10.7	24.1	20.0–28.3
No	2121 (95.2%)	62.2	61.3–63.0	4.3	4.1–4.5	9.9	9.4–10.4	23.6	22.8–24.4
Current alcohol consumption									
Yes	168 (7.6%)	61.6	58.5–64.7	4.7	3.9–5.5	10.2	8.5–11.9	23.6	20.6–26.6
No	2056 (92.4%)	62.3	61.4–63.1	4.3	4.1–4.5	9.8	9.3–10.3	23.6	22.8–24.5
Season of the year									
Summer	566 (25.3%)	63.4	61.8–65.0	4.1	3.8–4.4	8.9	8.1–9.8	23.6	22.0–25.1
Autumn	625 (28.0%)	62.1	60.6–63.5	4.4	4.0–4.7	9.9	9.0–10.7	23.7	22.3–25.1
Winter	501 (22.4%)	61.5	59.9–63.1	4.3	3.9–4.7	10.9	9.9–11.9	23.4	21.9–24.9
Spring	543 (24.3%)	60.7	59.0–62.3	4.3	3.9–4.6	9.9	8.9–10.8	25.2	23.6–26.8
Day of the week									
Weekdays	2091 (93.6%)	62.0	61.2–62.8	4.2	4.1–4.4	9.9	9.5–10.4	23.8	23.1–24.6
Weekend	144 (6.4%)	61.2	57.9–64.4	4.4	3.6–5.3	9.0	7.3–10.7	25.4	22.4–28.5

^1^Values with different superscript letters (a, b) indicate significant differences (*p* < 0.05) between groups of each NOVA classification.

^2^In 2018, 1 Brazilian Real (R$) was equivalent to 3.87 US dollars (US$).

## Data Availability

The data that support the findings of this study are available on request from the corresponding author. The data are not publicly available due to ethical restrictions.

## Supporting Information

Table 1S: Average contribution (%) of each NOVA classification food groups to total energy intake of pregnant women, according to the research center, EMDI-Brazil, 2021.
