# Supplementary material for: Consumption of Ultraprocessed Foods Among Brazilian Pregnant Women Attended in Primary Healthcare
Source: J Nutr Metab. 2025 Jun 2;2025:4538910. doi: 10.1155/jnme/4538910 (PMC12149507; doi:10.1155/jnme/4538910)
Supplement: Supporting Information — Additional supporting information can be found online in the Supporting Information section. [file 4538910.f1.docx]

**Table 1S.** Average contribution (%) of each NOVA classification food groups to total energy intake of pregnant women, according to research center, EMDI-Brazil, 2021.

| **Research centre** | **n** | **Unprocessed or minimally processed foods** | **Processed culinary ingredients** | **Processed foods** | **Ultra-processed foods** |
| --- | --- | --- | --- | --- | --- |
| Total | 2247 | 62,1 (61,3-62,9) | 4,3 (4,1-4.4) | 9,8 (9,4-10,3) | 23,8 (23,1-24,6) |
| Aracaju | 261 | 62,2 (59,9-64,4) | 4,4 (3,9-4,9) | 13,5 (12,1-15,0) | 19,9 (17,9-21,9) |
| Belo Horizonte | 181 | 59,3 (56,5-62,0) | 4,1 (3,4-4,7) | 10,7 (9,1-12,3) | 25,9 (23,4-28,5) |
| Brasília | 143 | 64,0 (60,9-67,2) | 3,7 (3,0-4,3) | 8,8 (7,1-10,5) | 23,5 (20,5-26,5) |
| Macaé | 218 | 60,3 (57,8-62,7) | 4,9 (4,3-5,4) | 10,1 (8,8-11,5) | 24,7 (22,3-27,2) |
| Palmas | 89 | 70,9 (67,5-74,3) | 2,7 (2,2-3,1) | 4,3 (2,8-5,7) | 22,1 (18,6-25,6) |
| Pinhais | 272 | 54,7 (52,6-56,8) | 5,0 (4,4-5,6) | 11,2 (9,8-12,5) | 29,1 (27,0-31,3) |
| Ribeirão Preto | 259 | 59,6 (57,5-61,8) | 3,9 (3,3-4,4) | 8,6 (7,5-9,7) | 27,9 (25,8-30,0) |
| Rondonópolis | 219 | 64,8 (62,4-67,3) | 3,8 (3,3-4,3) | 7,8 (6,6-9,1) | 23,6 (21,2-25,9) |
| São Luís | 292 | 66,5 (64,4-68,6) | 3,8 (3,4-4,2) | 9,1 (8,1-10,2) | 20,6 (18,6-22,5) |
| Viçosa | 254 | 64,9 (62,9-66,9) | 4,8 (4,4-5,3) | 9,7 (8,5-10,8) | 20,6 (18,6 -22,6) |
| Vitória | 46 | 59,2 (54,3-64,1) | 5,1 (3,5-6,6) | 11,9 (8,5-15,3) | 23,8 (18,7-28,9) |
